# Modulation of Fibroblast Growth Factor Signaling Is Essential for Mammary Epithelial Morphogenesis

**DOI:** 10.1371/journal.pone.0092735

**Published:** 2014-04-09

**Authors:** Xiaohong Zhang, Guijuan Qiao, Pengfei Lu

**Affiliations:** Wellcome Trust Centre for Cell Matrix Research, Faculty of Life Sciences, University of Manchester, Manchester, United Kingdom; II Università di Napoli, Italy

## Abstract

Fibroblast growth factor (FGF) signaling is essential for vertebrate organogenesis, including mammary gland development. The mechanism whereby FGF signaling is regulated in the mammary gland, however, has remained unknown. Using a combination of mouse genetics and 3D ex vivo models, we tested the hypothesis that *Spry2* gene, which encodes an inhibitor of signaling via receptor tyrosine kinases (RTKs) in certain contexts, regulates FGF signaling during mammary branching. We found that *Spry2* is expressed at various stages of the developing mammary gland. Targeted removal of *Spry2* function from mammary epithelium leads to accelerated epithelial invasion. *Spry2* is up-regulated by FGF signaling activities and its loss sensitizes mammary epithelium to FGF stimulation, as indicated by increased expression of FGF target genes and epithelia invasion. By contrast, *Spry2* gain-of-function in the mammary epithelium results in reduced FGF signaling, epithelial invasion, and stunted branching. Furthermore, reduction of *Spry2* expression is correlated with tumor progression in the MMTV-PyMT mouse model. Together, the data show that FGF signaling modulation by *Spry2* is essential for epithelial morphogenesis in the mammary gland and it functions to protect the epithelium against tumorigenesis.

## Introduction

Branching morphogenesis is a fundamental process whereby a cell or a group of cells expand their surface area by forming cellular or tissue extensions during development [1]. Many invertebrate and vertebrate organs, including fly trachea and the mammalian lung, kidney, and mammary gland, undergo branching morphogenesis as an essential part of their ontogeny [2]. Unlike most other vertebrate organs, however, mammary branching occurs primarily during postnatal development in the mouse [3]. Specifically, a primitive ductal epithelial tree undergoes rapid epithelial invasion into the stroma fat-pad with concurrent bifurcation of the terminal end bud (TEB) at the tip of each primary duct starting at 3 weeks of age at puberty onset. The process persists for another ∼6–7 weeks until primary ducts have extended to the distal end of the fat-pad and the TEBs regress. In addition to the primary ducts, the mammary epithelial tree is also elaborated by formation of lateral branches that sprout from the trailing primary ducts as well as tertiary side-branches until an intricate epithelial network emerges from the adult gland [4], [5].

A major focus in mammary gland biology has been to understand the cellular and molecular basis of epithelial branching morphogenesis. Several major signaling pathways, including Hedgehog [6], WNT [7], TGF-β [8], and integrin-extracellular matrix signaling [9], play positive or negative roles in regulating this intricate process. Another major pathway essential for mammary gland biology is signaling via receptor tyrosine kinases (RTKs)[10], [11]. RTK signaling is an ancient cell communication pathway and the RTK super-family is composed of a myriad of members, including those in the epidermal growth factor receptor (EGFR), the fibroblast growth factor receptor (FGFR)[12], and the insulin growth factor receptor (IGFR) families.

Regulation of RTK signaling is essential for normal development of the mammary gland. By contrast, RTK deregulation, resulted from either too little or too much signaling activities, leads to profound defects in normal development and can cause breast cancer. For example, reduction of FGF signaling due to loss of either *Fgf10* or its receptor *Fgfr2* causes a failure of mammary placode formation during embryogenesis [13], [14]. When FGF signaling is reduced during postnatal development due to conditional removal of *Fgfr1* or *Fgfr2*, epithelial branching is severely affected [15], [16]. Likewise, when EGF signaling is reduced due to removal of ligands in the EGF family including EGF, TGF-α, and amphiregulin, epithelial branching is greatly stunted [17]. Consistent with these data, mammary fat-pads lacking *Egfr* function fail to support epithelial outgrowth and branching [18], suggesting that EGF signaling targets the stroma and is essential for mammary gland branching.

Conversely, excessive RTK signaling has long been associated with breast cancer. For example, *ErbB2* upregulation promotes breast tumorigenesis and treatments aiming at blocking *ErbB2* function have remained an effective therapy against human breast cancer [19]. Likewise, excessive FGF signaling due to overactive FGF ligand or receptor causes breast tumors in vitro and in mouse models [20], [21], [22], [23]. Moreover, *Fgfr2* upregulation as a result of allelic polymorphism has been associated with human breast cancer [24], [25], suggesting a causal role of excessive *Fgfr2* activities in the disease.

One effective mechanism whereby RTK signaling is regulated depends on members of the *Sprouty* gene family, which contains four members *Spry1–4* in the mouse [26], [27]. As intracellular inhibitors of RTK signaling, *Spry2* and its family members play an essential role in numerous vertebrate developmental processes, including development of the tooth, cerebellum, and nervous system [28], [29], [30]. Importantly, *Spry2* gene is greatly down-regulated in subgroups of breast cancer, suggesting that it protects mammary epithelium from tumorigenesis [31], [32], [33]. In this study, we hypothesized that *Spry2* regulates RTK signaling in normal mammary gland development. To test this hypothesis, we analyzed the defects in mice lacking or overexpressing *Spry2*.

## Materials and Methods

### Mouse Strains

All of the mouse strains used in this study were maintained on a mixed genetic background and housed in a 12/12 hour light/dark cycle with food and water provided ad libitum in accordance with the Animal (Scientific Procedures) Act, 1986 (UK), project licence PPL 40/9865 and approved by the University of Manchester Ethical Review Process Committee. Mice carrying the *Spry2* ^fl^ [34] and *Spry2-*GOF [35] alleles were provided by Ophir Klein and were maintained on the (FvB/N; C57/BL6; 129Sv; Swiss black) background and the (FvB/N; C57/BL6) background, respectively. Those carrying the murine mammary tumor virus (MMTV)-Cre transgene D line [36] and the *R26R* ^fl^ Cre-reporter line [37] were purchased from the Jackson Laboratory. Offspring from crosses of the various lines were genotyped according to methods in the publications describing the mouse lines.

### Mammary Gland Wholemount Preparation, Photography and Morphometric Analysis

Mice were sacrificed by cervical dislocation or asphyxiation. Mammary glands were harvested and mounted on glass slides. They were stained with Carmine red and cleared as previously described [1]. Wholemount images were captured on a Zeiss Lumar dissection scope. Adobe Photoshop CS4 was used to process images and to measure length of epithelial ducts and branch points. The number of branch points per millimeter of duct was the mean number of branch points on three longest primary ducts divided by their mean length.

### Histology, Glycogen, and Lipid Staining of the Mouse Mammary Gland

Energy storage, including glycogen and lipids in the liver, of 12-week-old mice was measured as follows: liver was harvested, fixed in 4% paraformaldehyde, sectioned in paraffin at 5 μm of thickness, oxidized in 0.5% periodic acid for 5 minutes, rinsed in distilled water, and stained in Schiff reagent for 15 minutes. Sections were then washed in tap water for 5 minutes before counter-stained in hematoxylin. For the Oil-Red-O staining, fixed liver was embedded in OCT and frozen sectioned at 10 μm of thickness. Sections were rinsed in 60% isopropanol, stained in freshly prepared Oil-Red-O for 15 minutes, and rinsed again in 60% isopropanol. Hematoxylin was used to counter-stain sections. Interscapular, reproductive, and liver white adipose tissues were fixed in paraformaldehyde, embedded in paraffin and sectioned at 5μm thickness, and stained with hematoxylin and eosin.

### Preparation of Mammary Gland Epithelial Cells, Infection by Adenovirus

Donor mammary glands were harvested, minced, and dissociated in buffer [10 mM Hepes buffer, 5% fetal bovine serum (FBS), DMEM/F12, Penicillin-Streptomycin 100 U/ml] containing collagenase (Sigma C5138–1G, 2 mg/ml) for 1 hr at 37°C. Primary epithelial “organoids” were purified by five repetitions of washes in the dissociation buffer containing no collagenase and collected using a swing-bucket centrifuge at 400×*g*. Purified primary mammary epithelium was resuspended in growth medium (5 μg/ml insulin, 1 μg/ml hydrocortisone, 10 ng/ml EGF, 10% FBS, Penicillin-Streptomycin 100 U/ml, Gentamicin 50 μg/ml in DMEM/F12) and infected overnight with Adenovirus-Cre-GFP (green fluorescent protein) [38] at a multiplicity of infection of ∼25 particles per cell. The next day, organoids were washed several times with PBS and were cultured for another 24 hr to allow for recovery from infection before further manipulation.

### Fluorescence Activated Cell Sorting (FACS) and Quantitative Real Time PCR

For FAC sorting or analysis, single cells were dissociated and were fluorescently labeled by antibody staining or infection by Ad-Cre-GFP. Sorting was done using an Aria system and analysis using FACalibur system. Data were processed using FACS Diva software (BD Biosciences).

qPCR was performed using the 7500 Fast Real Time PCR system (Applied Biosystems) and data were normalized to expression of at least two of the reference genes, including *Actb*, *18S*, *Eef1g*, *Gapdh*. Primer sequences were described in Table S1.

### Assays for β-GAL and Human Placental Alkaline Phosphatase (PLAP) Activity

For β-GAL analysis, wholemount mammary glands were harvested, fixed for 30 min in 4% paraformaldehyde at room temperature, washed thoroughly in phosphate-buffered saline (PBS), and stained overnight in *LacZ* (which encodes β-GAL) staining buffer (Roche) at 37°C. For PLAP staining, fixed mammary glands were heated at 70°C for 30 minutes to inactivate endogenous alkaline phosphatases and stained in BM purple (Roche) overnight at 37°C.

### In Vitro Epithelial Branching and Invasion Assays

Either mammary organoids or MEC aggregates were used for branching and invasion assays. To aggregate MECs, sorted cells were pelleted, cultured via the “hanging-drop” method whereby a 50-μl drop of growth medium containing single cells was cultured upside-down on the lid of a petri dish overnight at 37°C. MEC aggregates were then washed in DMEM/F12 to eliminate fetal serum. For branching assay, basal medium containing growth factors FGF2 was used and was found to be a quantitative assay. Note that the branching kinetics differ somewhat depending on the mouse strains used.

For epithelial invasion assay, heparin acrylic beads of ∼100 μm in diameter were pre-soaked in bovine serum albumin (BSA) or FGF10 (100 μg/ml) overnight at 4°C. They were washed in PBS before use. Delivery of control epithelium (wild-type treated with Ad-Cre-GFP), beads, and experimental epithelium (*Spry2* ^fl/fl^ or *Spry2*-GOF treated with Ad-Cre-GFP), which was done sequentially, and sample positioning where they were kept ∼100 μm apart using a Tungsten needle were performed under a Zeiss stereoscope. Culture chamber containing Matrigel was chilled on ice during the experimental procedure to keep Matrigel from solidifying. After positioning, culture chamber was put on a 37°C heat block to speed up the gelling process. Basal medium was then added to samples before they were cultured 37°C.

### Data Mining of Expression Microarray

Microarray data were from the NCBI Gene Expression Ominbus under the accession numbers GSE2988, GSE5602, GSE5223, and GSE5221 [39], [40]. *Spry2* expression was determined by calculating M  =  log_2_(Cy5/Cy3), where Cy5 values of TEB/ductal epithelium from normal female mice at 5-weeks age, or hyperplasia and advanced carcinoma from PyMT female mice were compared with Cy3 values of the distal stromal from female mice at 5-weeks of age.

## Results

### *Spry2* Null Mice Show Stunted Epithelial Branching Likely due to Malnourishment

**Figure 1:**
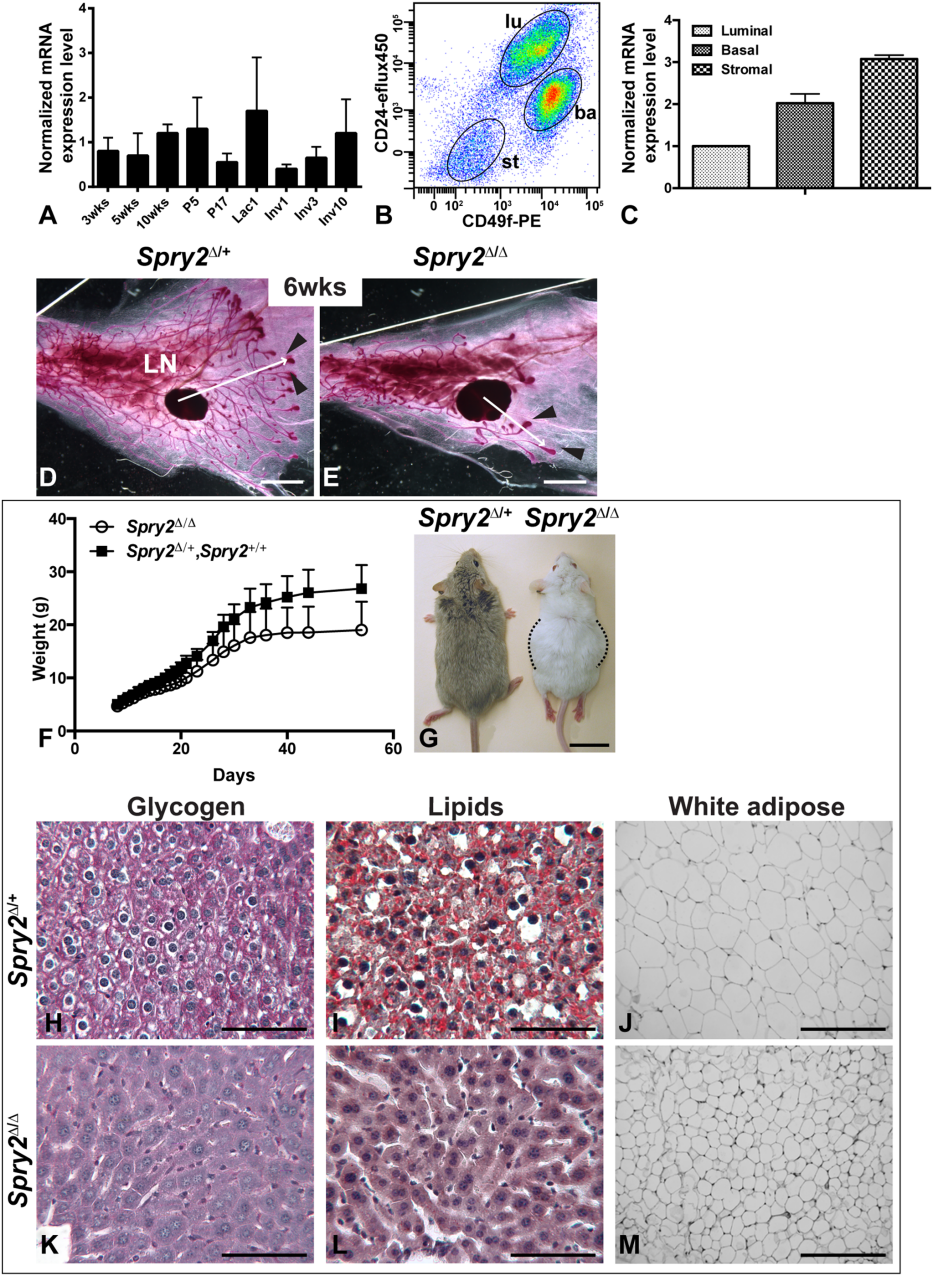
*Spry2* null mice show stunted epithelial branching due to malnourishment. (**A–C**) *Spry2* mRNA expression as detected by quantitative RT-PCR (qPCR). (**A**) *Spry2* mRNA expression was measured by qPCR using RNA harvested from mammary glands from female mice at 3-weeks, 5-weeks, and 10-weeks of age as virgins, during pregnancy (P) on day 5 and 17, on day 1 of lactation (Lac), and on day 1, 3, and 10 of involution (Inv). *Spry2* expression at 3-weeks was set as base value against which other stages were compared. Abbreviations: wks, weeks; P, pregnancy; L, lactation; Inv, involution. (**B**, **C**) MECs were sorted based on their expression of CD24 and Integrin-α6 (CD49f). CD24^med^CD49f^hi^ cells were basal (ba), whereas CD24^hi^CD49f^l^°^w^ cells and CD24^l^°^w^CD49f^l^°^w^ were luminal (lu) and stromal (st), respectively. RNA was harvested from the three cell partitions to generate DNA templates for qPCR reactions (**C**). (**D**, **E**) The mammary branching tree at 6-weeks of age, as revealed by Carmine Red staining of glands in wholemount. Proximal (close to the nipple) is to the left and distal is to the right. Arrowheads indicate TEBs at the tips of invading mammary epithelium, which persist until branching morphogenesis ceases in adult glands. Arrows indicate the extent of ductal penetration in the fat pad. Note epithelial branching was severely stunted in (**E**) mutant (*Spry2* ^Δ/Δ^; n = 8) mice when compared with (**D**) control (*Spry2* ^Δ/+^; n = 12) mice. Scale bars: 2 mm. Abbreviation: epi, epithelium; st, stroma; LN, lymph node. (**F–M**) *Spry2* null mice showed growth retardation (**F**, **G**) and an insufficiency in energy storage (**H–M**). (**F**) Growth curve of pups born from *Spry2* ^Δ/+^ crosses. Weights between *Spry2* ^Δ/+^ (n = 15) and *Spry2* ^+/+^ (n = 4) mice were indistinguishable and combined. Values shown are the mean ± SD for each data point. (**G**) Dorsal view of typical appearances of *Spry2* ^Δ/+^ and *Spry2* ^Δ/Δ^ mice at 12-weeks of age. Note *Spry2* ^Δ/Δ^ mice were shorter than normal and had enlarged midsection (flanked by dotted black lines) due to distended intestines (not shown). Scale bars: 2 cm. (**H–M**) Glycogen and lipid storage, as revealed by Periodic Acid-Schiff and Oil-Red-O staining, respectively, and histology of white adipose tissue from *Spry2* ^Δ/+^ (**H**–**J**) and *Spry2* ^Δ/Δ^ mice (**K**–**M**). Note that *Spry2* ^Δ/Δ^ mutant liver lacked glycogen (**K**) and lipid storage (**L**) as was evident in control liver (purple-magenta color in **H** and red droplets in **I**); moreover, adipocytes from white adipose tissue in *Spry2* ^Δ/Δ^ mutant (**M**) mice were smaller than normal (**J**). Scale bars: 100 μm.

We first examined expression of *Spry2* during various stages of postnatal mammary gland development using quantitative real-time PCR (qPCR). *Spry2* mRNA was readily detected at all the stages examined, including during epithelial branching, pregnancy, lactation, and involution stages (Fig. 1A). To examine the cell types that express *Spry2*, we used fluorescence-activated cell sorting (FACS) and sorted mammary cells based on their expression of cell surface markers of CD24 and CD49f (Integrin-α6) (Fig. 1B). We found that both epithelial cells, including luminal (CD24^hi^CD49f^l^°^w^) and basal (CD24^med^CD49f^hi^) cells, and stromal cells (CD24^l^°^w^CD49f^l^°^w^) readily expressed *Spry2* mRNA (Fig. 1C).

To determine whether the mammary gland develops normally in mice lacking *Spry2* function, we self-crossed *Spry2* heterozygous control mice (*Spry2* ^Δ/+^) and generated *Spry2* null mutant mice (*Spry2* ^Δ/Δ^). We found that the mammary gland formed in the *Spry2* mutant mice (Fig. 1D, E); however, in pubertal mice at 6 weeks, when vigorous mammary branching is ongoing, we noticed a defect in the branching tree in mutant glands (Fig. 1D, E; n = 8). In comparison to control glands, epithelial branching in mutant glands was greatly stunted: many fewer branches and terminal end buds (TEBs) were formed, and epithelial invasion into the stroma was greatly reduced. A similar phenotype of retarded epithelial branching was also observed at other pubertal stages, including at 5- and 7-weeks of age.

Moreover, mammary glands in mutant mice were considerably smaller than normal (Fig. 1D, E), suggesting the above mammary phenotype may result from a systemic defect in mutant mice. To further examine this possibility, we monitored the growth of *Spry2* mutant mice. We found that *Spry2* mutant pups were born at a Mendelian ratio (26%, n = 11/42; Table 1). By weaning at 21 days, almost half of the mutant pups had died (13.9% instead of the expected frequency at 25%; Table 1). When the pups from *Spry2* ^Δ/+^ self-crosses were weighed daily from day 8 to day 21, and then every 2–4 days up to 54 days, we found that *Spry2* mutant pups gained weight considerably more slowly at all of the stages examined (Fig. 1F). By 12 weeks, *Spry2* adult mutant mice were shorter and skinnier than normal (Fig. 1G) and had little energy storage, as evident from the lack of glycogen (Fig. 1H, K) and lipids (Fig. 1I, L) in their livers and the shrinkage of adipocytes in their white adipose tissues (Fig. 1J, M). Together, these data show that *Spry2* mutant mice are growth-retarded, a systemic defect that most likely leads to a secondary branching phenotype shown in the mutant mammary glands.

**Table 1:** Pups generated from self-crosses of *Spry2* heterozygous mice.

	*Spry2* ^+/+^		*Spry2* ^Δ/+^		*Spry2* ^Δ/Δ^	
Age	Act.	Exp.	Act.	Exp.	Act.	Exp.
P1	8 (19%)	10 (25%)	23 (54.8%)	21 (50%)	11 (26.2%)	11 (25%)
P21	91 (25.3)	90 (25%)	218 (60.7%)	179 (50%)	50 (13.9%)	90 (25%)

Pups were genotyped on postnatal day 1 (P1; n = 42) and upon weaning on postnatal day 21 (P21; n = 359). Note the actual frequencies (Act.) of both *Spry2* ^Δ/+^ and *Spry2* ^+/+^ were more than the expected frequencies (Exp.) because a portion of the *Spry2* ^Δ/Δ^ pups died prior to weaning.

### MMTV-Cre-mediated Conditional Removal of *Spry2* Function from Mammary Epithelium Causes Accelerated Epithelial Invasion

**Figure 2:**
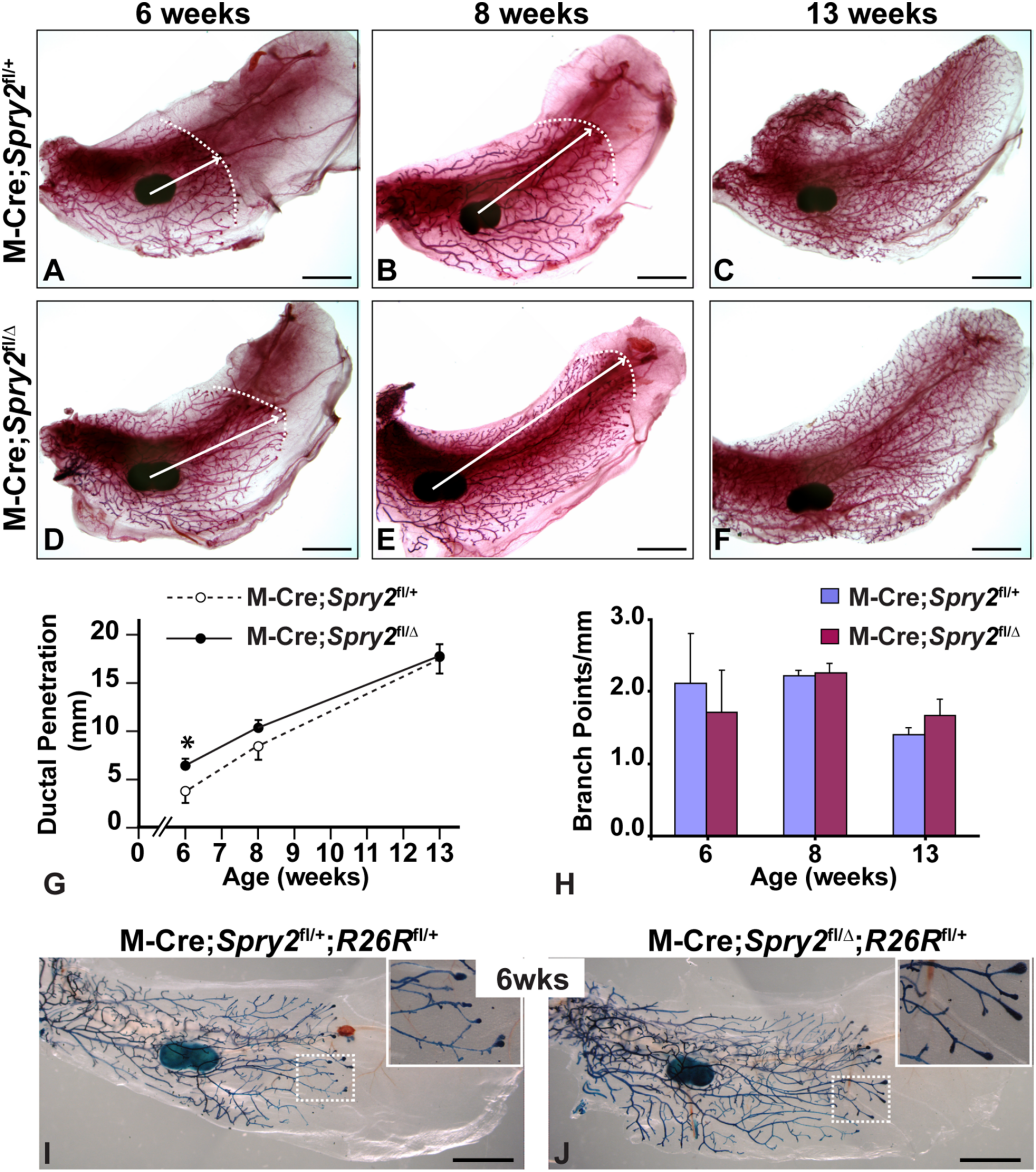
Conditional removal of *Spry2* function from mammary epithelium causes accelerated epithelial invasion. (**A–H**) The mammary branching tree from the #4 glands at the postnatal stages indicated, as revealed by Carmine Red staining of glands in wholemount. (**A–C**) glands from control (M-Cre;*Spry2* ^fl/+^) mice; (**D–F**) glands from mutant (M-Cre;*Spry2* ^fl/Δ^) mice. (A–F) Arrows indicate the extent of ductal penetration in the fat pad. Dotted white line illustrates the epithelial invasion front. (**G, H**) Quantitative comparisons of ductal penetration and branch point formation between control and mutant glands. At 6 weeks, ductal penetration measurements were 3.8±1.3 (control, n = 10) and 6.5±0.6 (mutant, n = 6); at 8 weeks, the measurements were 8.5±1.5 (control, n = 4) and 10.3±0.8 (mutant, n = 6); at 13 weeks, they were 17.4±1.5 (control, n = 8) and 17.8±1.1 (mutant, n = 14). Measurements of branching points were 2.1±0.7 (control) and 1.7±0.6 (mutant) at 6 weeks, 2.2±0.1 (control) and 2.2±0.2 (mutant) at 8 weeks, and 1.4±0.1 (control) and 1.7±0.2 (mutant) at 13 weeks. Values shown are the mean ± SD for each data point: *, P<0.05, unpaired, two-tailed Student’s *t* test. N is the number of mammary glands examined. (**I**, **J**) Assays for β-GAL activity in wholemount of control (**I**, M-Cre;*Spry2* ^fl/+^;*R* ^fl/+^) and mutant (**J**, M-Cre;*Spry2* ^fl/Δ^;*R* ^fl/+^) glands at 6-weeks of age. The dashed boxes demarcate the portions of branching trees that are shown at higher magnification in insets. β-GAL expression marks cells derived from those in which MMTV-Cre-mediated recombination occurred. Note that β-GAL-positive *Spry2* null cells were well represented in the distal branching network, including TEBs of mutant glands (**J**, n = 18). Scale bars: 2.5 mm.

To circumvent the growth retardation presented in *Spry2* conventional knockout mice, we used a conditional approach to eliminate *Spry2* function via Cre-mediated recombination based on the MMTV-Cre transgene (M-Cre) [36]. Male mice carrying one copy of M-Cre and heterozygous for a *Spry2* null allele, *Spry2* ^Δ^, were crossed with females homozygous for a *Spry2* conditional allele, *Spry2* ^fl/fl^ to generate control (M-Cre; *Spry2* ^fl/+^) and mutant (M-Cre; *Spry2* ^fl/Δ^) female mice. We then examined mammary gland development in these animals at several critical stages (Fig. 2).

At 3 weeks after birth, we observed a rudimentary ductal tree in the mutant glands (not shown; n = 4) that was not obviously different from that observed in control glands. However, in pubertal mice at 6 weeks, when vigorous mammary branching is occurring, we noticed a defect in the branching tree in mutant glands (Fig. 2A, D). In comparison to control glands, mammary ducts in mutant glands penetrated 71% more than the normal distance into the fat pad (Fig. 2G), however they did not form a significantly fewer number of branch points per millimeter (Fig. 2H). Ductal penetration was also increased by 21% in the mutants when compared with control glands at 8 weeks (Fig. 2B, E). By 13 weeks, both control and mutant glands were completely infiltrated by ductal epithelium and the epithelial trees overall were similar in control and mutant mice (Fig. 2C, F). Together, the above data suggest that conditional removal of *Spry2* function from mammary epithelium causes accelerated epithelial invasion.

Previous studies have shown that M-Cre functions incompletely in the mammary epithelium, leading to the possibility that Cre-expressing cells are out-competed by control cells and excluded from the distal epithelial network [15]. To examine where *Spry2* mutant cells were distributed in the epithelial network, we employed a reporter allele, *R26R* ^fl^, that expresses *lacZ* when it has undergone Cre-mediated recombination [37]. We first produced a line of mice homozygous for both *Spry2* ^fl^ and *R26R* ^fl^ (*Spry2* ^fl/fl^;*R* ^fl/fl^), and then crossed females of this line with male mice carrying M-Cre and heterozygous for the *Spry2* ^Δ^ allele, to generate control (M-Cre;*Spry2* ^fl/+^;*R* ^fl/+^ *)* and mutant (M-Cre;*Spry2* ^fl/Δ^;*R* ^fl/+^) progeny. Next, we assayed the mammary glands from control and mutant mice for β-GAL activity at various postnatal stages to determine the distribution of cells in which Cre-mediated recombination had occurred. At 6 weeks, β-GAL-positive cells were evenly dispersed throughout the entire branching network, including distal ductal epithelium and TEBs, in both control and mutant glands (Fig. 2I, J). Similar observations were also made on mammary glands in older mice, including at 7 or 9 weeks of age. Together, these data indicate that Cre-expressing *Spry2* null cells in mutant glands are not excluded from the distal epithelial network and suggest that they are most likely not out-competed by Cre-negative control cells in the mosaic gland.

### *Spry2* Null Epithelium Shows Enhanced FGF Signaling and Increased Epithelial Branching Activities in vitro

**Figure 3:**
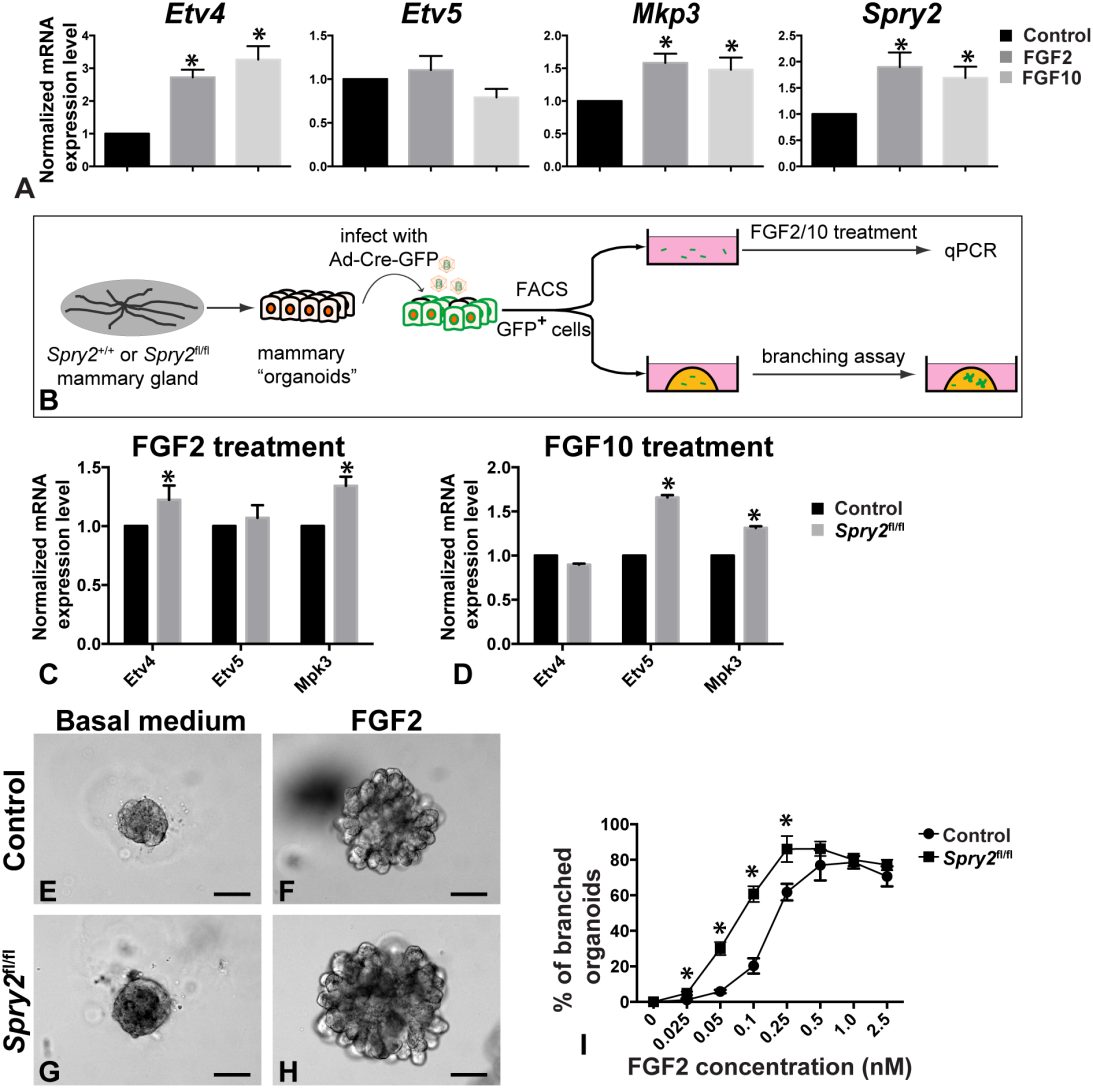
*Spry2* null epithelium shows enhanced FGF signaling activities and increased epithelial branching activities. (**A**) Expression, as measured by qPCR, of *Spry2* and target genes of FGF signaling, including *Etv4*, *Etv5*, and *Mkp3*, in response to a 24-hour treatment of FGF2 (10 nM) or FGF10 (10 nM). Expression is relative to that of the untreated samples. Values shown are the mean ± standard deviation (SD) of three independent experiments. Statistically significant differences of p<0.05 (t test) were observed between expression of untreated and treated samples for all genes except for *Etv5* in response to FGF2 and FGF10 treatment. (**B**) Schematic diagram depicting the experimental procedure in sample preparation, treatment, and analysis. Mammary organoids were prepared from *Spry2* ^+/+^ and *Spry2* ^fl/fl^ mice and were infected with adenovirus-Cre-GFP, which generated control (*Spry2* ^+/+^) and mutant (*Spry2* ^Δ/Δ^) organoids, respectively. Transduced cells were then purified by FACS based on their expression of GFP before they were subjected to analyses on gene expression and epithelial morphogenesis in the presence or absence of FGF2 or FGF10. (**C–D**) Expression, as measured by qPCR, of *Etv4*, *Etv5*, and *Mkp3* in control and mutant MECs in response to 24-hour treatment of FGF2 (200 ng/ml, **C**) or FGF10 (200 ng/ml, **D**). Expression is relative to that of the control samples. Statistically significant differences of p<0.05 (t test) were observed between expression of control and mutant samples for all genes except for *Etv5* in response to FGF2 treatment and *Etv4* in response to FGF10 treatment. (**E–I**) in vitro branching assay in which control (**E**, **F**) and mutant organoids (**G**, **H**) were subjected to cultures in basal medium with (**F**, **H**) or without FGF2 (**E**, **G**). When stimulated by FGF2 at progressively higher concentrations from 0.025 nM to 0.5 nM, a progressively higher percentage of organoids underwent branching. At 1.0 nM and 2.5 nM, FGF2 did not stimulate a higher percentage of branched organoids to form. In addition to their differences in branching kinetics, *Spry2* ^Δ/Δ^ organoids overall formed larger branched structures than control organoids. Scale bars: 100 μm. (**I**) Quantitative comparisons of control and mutant MECs in their ability to undergo epithelial branching in vitro. Data were from experiments repeated three times or more. At least 100–150 organoids were examined for each treatment conditions. Values shown are the mean ± SD for each data point: *P<0.0005, unpaired, two-tailed Student’s *t* tests.

To determine whether *Spry2* is a negative feedback regulator of FGF signaling, which functions in the mammary epithelium [15], [16], we first assessed whether FGF stimulation can induce *Spry2* expression by mammary epithelial cells (MECs). As expected, treatment of MECs with either FGF2 or FGF10 caused an increased expression of FGF signaling targets *Etv4* and *Mkp3*, but curiously not *Etv5* (Fig. 3A) [41], [42]. We found that *Spry2* expression was upregulated by 90% and 69% by treatment with FGF2 and FGF10, respectively (Fig. 3A), suggesting that *Spry2* is a downstream target of FGF signaling in the mammary epithelium.

Next, we sought to determine how *Spry2* loss of function affects FGF signaling activities both on its target gene expression and epithelial morphogenesis. Mammary organoids were prepared from *Spry2* ^+/+^ and *Spry2* ^fl/fl^ mice and were infected with adenovirus-Cre-GFP, which generated control (*Spry2* ^+/+^) and mutant (*Spry2* ^Δ/Δ^) organoids, respectively. Adenovirus-transduced cells, which were GFP^+^, were then purified by FACS and subjected to analyses on gene expression and an in vitro branching assay (Fig. 3B). When compared with control MECs, mutant MECs showed a 23%, 7%, and 34% expression increase in *Etv4*, *Etv5*, and *Mkp3*, respectively, after FGF2 treatment (Fig. 3C). Likewise, mutant MECs showed a 66% and 31% expression increase in *Etv5* and *Mkp3*, respectively, after FGF10 treatment when compared with control MECs (Fig. 3D). These data show that overall MECs lacking *Spry2* function are sensitized to FGF signaling activities and *Spry2* is a negative regulator of FGF signaling in mammary epithelium.

To determine whether *Spry2* mutant epithelium undergoes branching morphogenesis more readily than normal, we turned to the FGF2-based 3D organotypic in vitro culture system that has been used for modeling epithelial branching [43](Fig. 3E–I). We found that mammary organoids formed branched structures at a progressively higher percentage when FGF2 was used at a progressively higher concentration until a plateau was reached (Fig. 3I). The quantitative nature of this assay thus allowed us to examine accurately how *Spry2* loss may affect the branching kinetics of mammary epithelium. We found that the branching kinetics differ somewhat depending on the mouse strains used (compare Fig. 3I with 5F). Interestingly, a higher than normal percentage of *Spry2* mutant organoids underwent branching at each of the concentrations before the plateau was reached (Fig. 3I). Moreover, branching structures from mutant MECs were noticeably bigger than normal (Fig. 3F, H). Together, these data demonstrate that *Spry2* mutant epithelium has a higher than normal level of FGF signaling and undergoes branching morphogenesis more readily than normal.

### MMTV-Cre-mediated *Spry2* Overexpression in the Mammary Epithelium Causes Retarded Epithelial Branching

**Figure 4:**
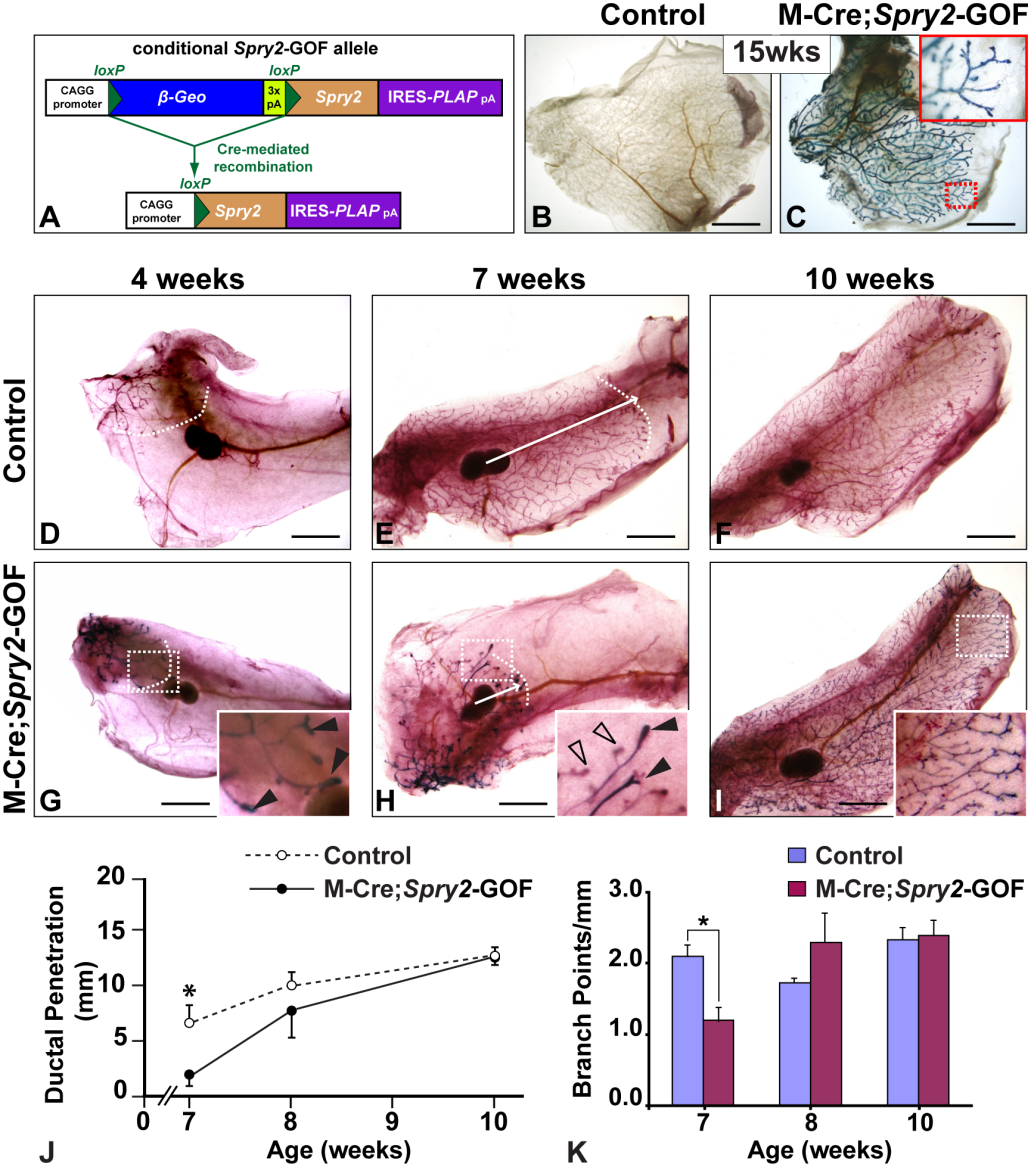
Gain of *Spry2* function in the mammary epithelium causes retarded epithelial branching. (**A**) Schematic diagram depicting the *Spry2*-GOF transgene. The β*-Geo* gene was driven by the CAGG promoter and followed by a triple poly-adenylation sequence (3x pA). Upon Cre-mediated recombination, the β*-Geo* gene was deleted and the mouse *Spry2* and human placental alkaline phosphatase (PLAP), constructed as a bi-cistronic mRNA containing an internal ribosome entry site (IRES) directing PLAP translation, were expressed. (**B–C**) Assay for PLAP activities in control (M-Cre or *Spry2*-GOF) and mutant (M-Cre;*Spry2*-GOF) #3 glands from adult female mice at 15-weeks of age. Note PLAP activities were detected in mutant (**C**) but not in control glands (**B**). The area in the dashed red box is highlighted in a close-up picture in the inset, illustrating the branching network that was positive for PLAP activities. (**D–I**) The mammary branching tree from #4 glands at the postnatal stages indicated. Samples were assayed for PLAP activities and were then stained with Carmine Red. (**D–F**) glands from control mice; (**G–I**) glands from mutant mice. Arrows indicate the extent of ductal penetration in the fat pad. Dotted white line illustrates the epithelial invasion front. Insets in (**G**), (**H**), and (**I**) show high-magnification views of the rudimentary ductal tree (area in dashed box), illustrating only some of the mammary epithelial cells showed PLAP activities due to the mosaic activity of the M-Cre transgene. Solid arrowheads indicate TEBs from 4-week (**G**) and 7-week (**H**) mammary glands that were more heavily stained for PLAP activities than other TEBs indicated by open arrowheads (**H**). These data suggest that the mammary glands from the bi-transgenic mice (M-Cre;*Spry2*-GOF) are mosaic, containing both Cre-expressing and non-Cre-expressing cells. (**J**, **K**) Quantitative comparisons of ductal penetration and branch point formation between control and mutant glands. At 7 weeks, ductal penetration measurements were 7.0±1.9 (control, n = 6) and 1.8±1.0 (mutant, n = 6); at 8 weeks, the measurements were 9.9±1.2 (control, n = 4) and 7.7±2.3 (mutant, n = 10); at 10 weeks, they were 12.4±0.3 (control, n = 8) and 12.7±0.04 (mutant, n = 4). Measurements of branching points were 2.1±0.3 (control) and 1.2±0.2 (mutant) at 7 weeks, 1.7±0.1 (control) and 2.3±0.4 (mutant) at 8 weeks, and 2.3±0.2 (control) and 2.4±0.2 (mutant) at 10 weeks. Values shown are the mean ± SD for each data point: *, P<0.05, unpaired, two-tailed Student’s *t* test. Scale bars: 2.5 mm. N is the number of mammary glands examined.

To reduce FGF signaling in the mammary epithelium, we employed a mouse line carrying a conditional *Spry2* gain-of-function allele, *Spry2-*GOF [35]. In these mice, transgene-harboring cells express β-GEO, a fusion protein with neomycin-resistance and β-galactosidase (β-GAL) activities [44] (Fig. 4A). Cells in which the transgene has undergone Cre-mediated recombination produce a bi-cistronic mRNA containing both *Spry2-* and human placental alkaline phosphatase (*PLAP*)-coding sequences (Fig. 4A). PLAP activity thus functions as a convenient reporter for the expression of the recombined transgene. To obtain mice in which the transgene was recombined in mammary epithelium, we crossed mice carrying *Spry2-*GOF and M-Cre. In adult mammary glands from the M-Cre;*Spry2-*GOF offspring at 15-weeks of age, we detected PLAP activity only in the epithelial network in which M-Cre is known to function (Fig. 4B, C).

Next, we examined mammary gland development in these animals at several critical stages (Fig. 4D–I). To compare the distribution between M-Cre;*Spry2-*GOF mutant cells (PLAP^+^ due to M-Cre expression) and *Spry2-*GOF control cells (PLAP^−^ due to lack of M-Cre expression) in the epithelial network of the mosaic glands, mammary glands were first assayed for PLAP activities before they were subjected to Carmine-Red staining. At 4 weeks after birth, pubertal epithelial branching had just started but ductal epithelium had yet to invade past the lymph node in both mutant and control glands (Fig. 4D, G). We observed a ductal tree in the mutant glands (M-Cre;*Spry2-*GOF, n = 6) that was not greatly different from that observed in control glands (M-Cre or *Spry2-*GOF, n = 6). Moreover, much of the mutant epithelial network was positive for PLAP staining (Fig. 4G).

At 7 weeks, we observed strong defects in the branching tree from mutant mammary glands (Fig. 4E, H). In comparison to control glands, mammary ducts in mutant glands penetrated 43% of the normal distance into the fat pad (Fig. 4J) and formed a slightly fewer number of branch points per millimeter (Fig. 4K). Moreover, the mammary epithelium showed an uneven staining of PLAP activity due to the presence of both M-Cre;*Spry2-*GOF positive cells and control cells (Fig. 4H). These data suggest that M-Cre;*Spry2-*GOF positive cells are less competitive than control cells when the epithelium undergoes a vigorous branching process. Ductal penetration was also reduced in the mutants at 8 weeks (Fig. 4J, K, and data not shown), although the epithelium was able to eventually fill the mammary fat-pad in mutant glands (Fig. 4I). By 10 weeks when epithelial branching had already finished in control glands (Fig. 4E), we found that mutant glands were completely infiltrated by ductal epithelium and the epithelial trees overall were similar in control and mutant mice (Fig. 4F, I–K). Together, these data suggest that a conditional gain of *Spry2* function in the mammary epithelium stunts branching morphogenesis.

### Gain of Spry2 Function in the Mammary Epithelium Reduces FGF Signaling Activities, Epithelial Invasion and Branching in Vitro

**Figure 5:**
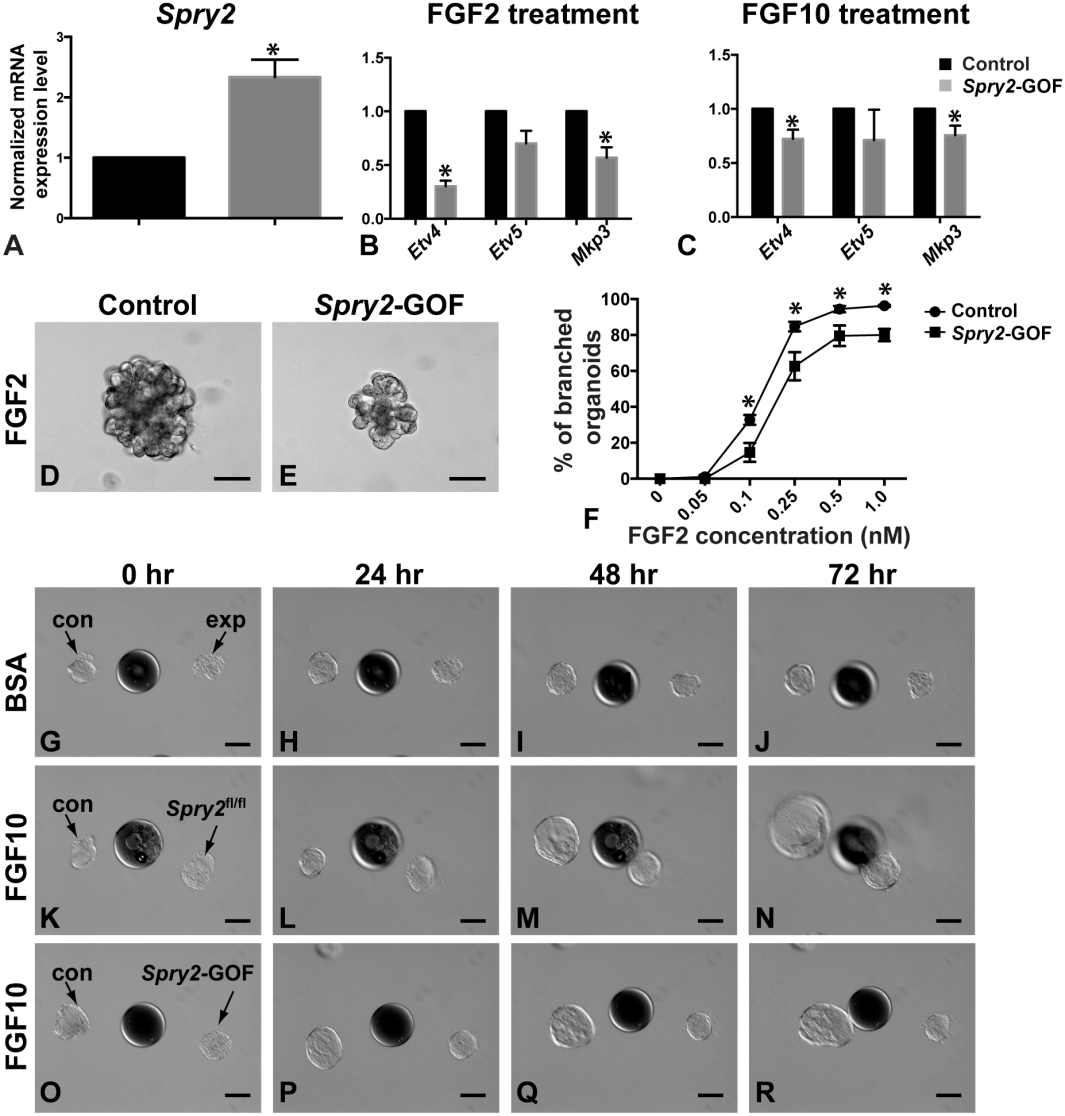
Gain of Spry2 function in the mammary epithelium reduces FGF signaling activities, epithelial invasion and branching in vitro. (**A**) *Spry2* expression, as measured by qPCR, in control and mutant MECs. MECs were prepared similar to the scheme described in Fig. 3B. Adenovirus-transduced control (*Spry2* ^+/+^) and mutant (*Spry2-*GOF) MECs were GFP^+^ and were purified by FACS. Purified cells were used for RNA harvest and qPCR assays. (**B–C**) Expression of the FGF signaling target genes *Etv4*, *Etv5*, and *Mkp3* in control and mutant organoids in response to 24-hour treatment of FGF2 (200 ng/ml, **B**) or FGF10 (200 ng/ml, **C**). Expression is relative to that of the control samples. Values shown are the mean ± standard deviation (SD) of three independent experiments. Statistically significant differences of p<0.05 (t test) were observed between expression of control and mutant samples for all genes except for *Etv5*. (**D–F**) in vitro branching assay in which control (**D**) and *Spry2-*GOF mutant organoids (**E**) were subjected to cultures in basal medium containing FGF2. When stimulated by FGF2 at progressively higher concentrations from 0.05 nM to 0.5 nM, a progressively higher percentage of MECs underwent branching, but a plateau was reached at 1.0 nM. In addition to forming branched structures at a lower than normal percentage, *Spry2-*GOF mutant structures were overall smaller than those derived from control MECs. Data were from experiments repeated three times or more. At least 100–150 organoids were examined for each treatment conditions. (**F**) Quantitative comparisons of control and mutant MECs in their ability to undergo epithelial branching in vitro. Values shown are the mean ± SD for each data point: *P<0.0005, unpaired, two-tailed Student’s *t* tests. Scale bars: 100 μm. (**G–R**) Mammary epithelial responses to beads pre-soaked in BSA (**G–J**) or FGF10 (**K–R**) during a 72-hour time course. Heparin acrylic beads of ∼100 μm in diameter were juxtaposed with mammary organoids at a distance of ∼100–150 μm. Control (con) organoids were on the left and experimental (exp) ones, including *Spry2* ^Δ/Δ^ (**K–N**) and *Spry2*-GOF ones (**O–R**), were on the right side of heparin beads. Note *Spry2* ^Δ/Δ^ organoids (n = 4) migrated faster than control ones and reached the bead by 48 hours (**K–N**) rather than 72 hours that controls took (**K–R**); by contrast, *Spry2*-GOF organoids (n = 5) moved much slower than control and had not reached FGF10-beads by 72 hours (**O–R**). Scale bars: 100μm.

To quantify the level of increase in *Spry2* expression in *Spry2-*GOF MECs, we infected mammary organoids from *Spry2* ^+/+^ and *Spry2-*GOF mice with adenovirus-Cre-GFP, which generated control (*Spry2* ^+/+^) and mutant (*Spry2-*GOF) organoids, respectively. Adenovirus-transduced cells, which were GFP^+^, were then purified by FACS and subjected to gene expression and functional analyses (Fig. 5). Using qPCR, we found that the level of *Spry2* RNA in *Spry2-*GOF cells were 2.3 times of that in control cells (Fig. 5A).

Next, we sought to determine how *Spry2* gain of function affects FGF signaling activities both on its target gene expression and epithelial morphogenesis. As noted before, FGF2 or FGF10 treatment upregulates expression of FGF signaling target genes (Fig. 3A). Interestingly, when compared to the control MECs that were treated with FGF2, mutant MECs showed a 70%, 30%, and 43% decrease in *Etv4*, *Etv5*, and *Mkp3* expression, respectively (Fig. 5B). Likewise, mutant MECs showed a 28%, 29%, and 24% decrease in *Etv4*, *Etv5*, and *Mkp3* expression, respectively, after FGF10 treatment when compared with control MECs (Fig. 5C). These data show that overall MECs overexpressing *Spry2* have a reduced level of FGF signaling in the mammary epithelium.

To determine the effect of reduced FGF signaling activities on epithelial branching, we subjected control and *Spry2-*GOF MECs to the aforementioned 3D in vitro assay based on FGF2 stimulation (Fig. 3E–I). As expected, control MECs formed branched structures at a progressively higher percentage when FGF2 was used at a progressively higher concentration until a plateau was reached. However, a lower than normal percentage of *Spry2* mutant organoids underwent branching at all of the concentrations tested (Fig. 5F). Moreover, branching structures from mutant MECs were considerably smaller than normal (Fig. 5D, E). These data thus are consistent with those from in vivo analysis (Fig. 4) and indicate that reduced FGF signaling due to *Spry2* overexpression inhibits epithelial branching in the mammary gland.

To understand how an increase or decrease of FGF signaling activities influences epithelial invasion, we examined the effects of *Spry2* loss or gain of function on migration toward beads pre-soaked in FGF10 during a 72-hour culture (Fig. 5G–R). Neither control nor experimental mammary epithelia, including both *Spry2* ^Δ/Δ^ and *Spry2-*GOF epithelia, invaded toward beads pre-soaked in bovine serum albumin (BSA) (Fig. 5G–J) or beads soaked in FGF2, which stimulate cyst formation (not shown), during the 72-hour time course. By contrast, both control and *Spry2* ^Δ/Δ^ epithelia responded to FGF10 and invaded toward beads presoaked in the protein. Interestingly, *Spry2* ^Δ/Δ^ epithelium invaded at a faster speed and closed the gap distance of 100–150 μm in 48 hours (Fig. 5K–N, n = 4) instead of 72 hours required by control epithelium (Fig. 5K–R, n = 9). By contrast, *Spry2-*GOF mutant epithelium barely invaded toward FGF10-beads and did not reach them by 72 hours (Fig. 5O–R, n = 5). These data thus corroborate our previous findings showing that FGF signaling is essential for epithelial invasion and show that *Spry2* loss- and gain-of-function accelerates and impedes epithelial invasion, respectively.

### *Spry2* Expression Is Reduced in the MMTV-PyMT Mouse Model

**Figure 6:**
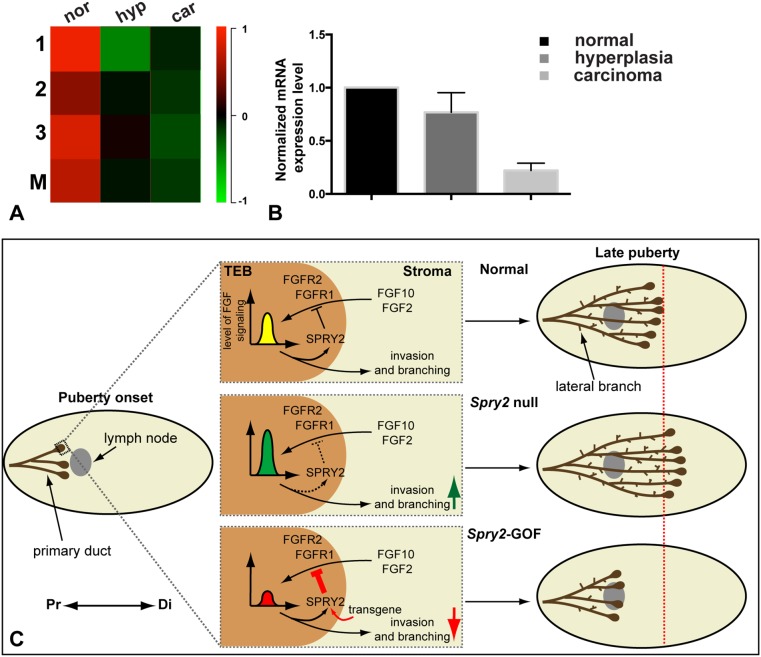
Spry2 expression is reduced in the MMTV-PyMT mouse model. (**A–B**) *Spry2* expression at different stages of cancer progression in the MMTV-PyMT mouse model. (**A**) *Spry2* expression in normal ductal epithelium (nor) and PyMT epithelium during the hyperplastic adenoma (hyp) and advanced carcinoma (car) stages relative to that in the distal un-invaded stroma of virgin female mice at 5-weeks of age. Analysis was based on data published in [39], [40]. Epithelium/distal stroma (Cy5/Cy3) expression ratios are shown for three independent experiments (lanes 1–3) and their respective means (M) using the color scale shown, with black indicating no difference in expression, red indicating relative enrichment in normal or cancer epithelium and green representing higher expression in the distal stroma. (**B**) *Spry2* mRNA expression was measured by qPCR using RNA harvested from mammary glands at the above stages. Values were normalized against actin expression and *Spry2* expression in normal glands was set as base value against which other stages were compared. (**C**) Model diagram depicting *Spry2* function in regulation of FGF signaling during mammary gland epithelial branching. The terminal end buds (TEBs) develop at the onset of puberty (three weeks after birth) at the distal tip of each primary duct. In the following six weeks the TEBs invade the stroma in a proximal (Pr)-to-distal (Di) direction until the whole fat-pad is occupied by around nine weeks of age. The epithelial network is further elaborated by lateral branches on the side of primary ducts. Regulation of FGF signaling levels is essential for normal epithelial branching morphogenesis in the mammary gland. Stromal FGF10 and FGF2 activate FGFR2 and FGFR1 in the epithelium and stimulate SPRY2 expression. SPRY2 in turn fine-tunes FGF signaling level (yellow bell-shape) and regulates epithelial invasion and branching into the stroma. In the absence of SPRY2 function in the mutant mammary glands, FGF signaling level (green bell-shape) is higher than normal due to the loss of a negative regulator. As a consequence, epithelial invasion into the fat-pad is accelerated (green arrow) during branching morphogenesis. Conversely, SPRY2 expression is augmented in *Spry2-*GOF mammary glands, due to intrinsic transcriptional regulation and expression from the transgene (curved red arrow) and FGF signaling level (red bell-shape) is reduced as a result. As a consequence, epithelial invasion is stunted (red arrow) during mammary branching at late puberty.

*Spry2* expression is greatly reduced in human breast cancer [31], [32], suggesting that it plays a role in protecting mammary epithelium against tumorigenesis. As an initial step in understanding *Spry2* function in breast cancer biology, we examined *Spry2* expression during tumor progression in the MMTV-PyMT mouse model of luminal B breast cancer [45]. We surveyed *Spry2* expression in an existing microarray expression database, in which gene expression of normal or cancer epithelium was compared with that of the distal stroma of 5-week-old virgin female mice [39]. We found that *Spry2* expression was readily detectable in ductal epithelium of normal glands (Fig. 6A). Interestingly, *Spry2* expression was greatly reduced in both hyperplastic adenoma and advanced carcinoma (Fig. 6A). To validate the data from microarray, we used qPCR and examined *Spry2* expression in hyperplasia and carcinoma from the PyMT model when compared with that in the normal epithelium (Fig. 6B). We found that *Spry2* RNA expression in hyperplasia and carcinoma was 77% and 22%, respectively, of that in the normal epithelium (Fig. 6B). Together, these data show that *Spry2* expression is lost during cancer progression and suggest that *Spry2* protects normal epithelium from breast cancer in the mouse mammary gland.

## Discussion

Despite its essential roles in mammary gland development and breast cancer, how RTK signaling is regulated in the mammary gland has remained largely unknown. Here, we show that *Spry2* is expressed in the epithelium and stroma at various stages of mammary gland development. Targeted removal of *Spry2* function from mammary epithelium leads to precocious epithelial invasion as a result of up-regulated FGF signaling activities and increased invasion of mutant epithelium. By contrast, *Spry2* overexpression in the mammary epithelium results in reduced FGF signaling and epithelial invasion, and consequently stunted branching morphogenesis. Moreover, *Spry2* expression is reduced the MMTV-PyMT mouse model of breast cancer. Together, these data show that FGF signaling modulation by *Spry2* is essential for normal epithelial morphogenesis in the mammary gland and suggest that its function is a protective mechanism against tumorigenesis.

### *Spry2* Is a Feedback Regulator of FGF Signaling in the Mammary Epithelium

The data showing that stimulation by either FGF2 or FGF10 induces *Spry2* expression suggest that *Spry2* is a downstream target of FGF signaling. Moreover, target genes of FGF signaling are upregulated in the absence of *Spry2* function and down-regulated when *Spry2* is overexpressed. Together, these data demonstrate that *Spry2* is a negative feedback regulator of FGF signaling, a major RTK pathway essential for mammary gland development and breast cancer biology.

An important question is whether *Spry2* regulates signaling via *Fgfr1* or *Fgfr2*, both of which play a role in postnatal branching morphogenesis [15], [16], [46]. Interestingly, studies have shown that FGF2 elicits a stronger mitogenic effect on cells expressing FGFR1 than those expressing FGFR2, whereas FGF10 does the opposite by triggers a stronger mitogenic effect on cells expressing FGFR2 than FGFR1 [47], [48]. These data suggest that FGF2 and FGF10 preferentially activate FGFR1 and FGFR2, respectively. Considering that *Spry2* null epithelium is sensitized to stimulation by both FGF2 and FGF10, it is likely that *Spry2* regulates signaling via both *Fgfr1* and *Fgfr2* in the mammary epithelium (Fig. 6C).

Our results do not exclude the possibility that *Spry2* regulates other RTKs, some of which, including ErBB2 and IGF1 receptor, are known to function in the mammary epithelium [49], [50]. Indeed, various RTKs, including FGFR, RET, and BDNF receptor, are regulated by *Sprouty* genes in different developmental contexts [28], [29], [30]. It thus remains an important future question as to what other RTKs may be regulated by *Spry2* in the mammary gland epithelium. Likewise, it also remains possible that *Spry2* functions in the mammary gland stroma. Our data from qPCR show that *Spry2* is expressed in the mammary stroma, where EGFR signaling is known to function [18].

Furthermore, our data do not differentiate what sub-branches of FGF signaling, including ERK, PI3K, and calcium signaling, are regulated by *Spry2* during mammary gland development. Although traditionally ERK signaling is better understood in mammary gland biology[43], [51], recent studies show that PI3K signaling also plays a role. Using cell lines of the mouse epithelium, Zhu and Nelson show that *Spry2* blocks PI3K signaling and represses epithelial branching in vitro [52]. These data, however, have yet to be substantiated by in vivo studies.

Together, the demonstration of *Spry2* function in the mammary gland accentuates a recurring theme in the field of developmental biology, which shows that most major signaling pathways are governed by negative feedback mechanisms. Such mechanisms reinforce the robustness of complex biological systems by allowing them better withstand fluctuations of essential intrinsic or extrinsic factors during development and homeostasis.

### Regulation of FGF Signaling by *Spry2* Is Essential for Epithelial Morphogenesis in the Mammary Gland

The level of FGF signaling needs to be tightly regulated to ensure normal development of the mammary gland. Deregulation of FGF signaling, whether as a reduction due to FGF ligand and receptor removal or as an increase due to an overactive signaling component, often leads to a failure of mammary gland development or breast tumor formation. In the current study, we show that one way whereby FGF signaling is regulated in the mammary epithelium is via *Spry2* gene function.

We show targeted removal of *Spry2* function from the epithelium causes precocious branching morphogenesis. Consistent with the in vivo phenotype, *Spry2* null epithelium is sensitized to FGF2 stimulation and forms branched structures more readily than normal. Likewise, *Spry2* null epithelium is sensitized to FGF10 stimulation and undergoes collective invasion more rapidly than normal. By contrast, *Spry2*-GOF epithelium is dampened to FGF2 stimulation and forms branched structures less readily than control epithelium. Furthermore, *Spry2*-GOF epithelium is desensitized to FGF10 stimulation and barely undergoes collective invasion. These data are consistent with the in vivo phenotype showing that targeted overexpression of *Spry2* in the mammary epithelium stunts epithelial invasion.

Taken together, these data suggest that regulation of FGF signaling by *Spry2* is essential for both FGF2-based ductal elongation and FGF10-mediated epithelial invasion during normal development. An increase of FGF signaling activities, for example due to *Spry2* loss, ductal elongation and epithelial invasion is more rapid than normal, which leads to accelerated epithelial invasion during pubertal branching. Conversely, a decrease of FGF signaling activities due to *Spry2*-GOF, ductal elongation and epithelial invasion is slower than normal, leading to a stunted epithelial invasion during postnatal branching of the mammary gland (Fig. 6C).

### Use 3D in vitro Models to Dissect Aspects of Epithelial Branching

The use of 3D in vitro cultures, including FGF2-based branching model, has greatly enhanced our understanding of epithelial morphogenesis in the mammary gland. Interestingly, although *Spry2* null MECs form branched structures more readily than normal, mammary glands lacking *Spry2* function show mostly accelerated epithelial invasion whereas branch formation is relatively not affected. Likewise, *Spry2-*GOF MECs form branches structures less readily than normal, yet epithelial invasion is more severely stunted than branch formation in mutant mammary glands.

It remains unclear the cause of this apparent discrepancy between in vitro and in vivo data. However, it highlights the importance of understanding precisely what aspects of epithelial branching that are modeled by a particular 3D culture. One possibility is that the FGF2-based culture system recapitulates more the cell behavior associated with ductal elongation, an essential step in epithelial invasion, than with that associated with branch-point formation. This notion is indeed supported by the observation that a high rate of cell proliferation exists during culture and, when it is inhibited, epithelial elongation fails to occur [51], [53].

By contrast, mammary epithelia lacking or over-expressing *Spry2* invade more quickly or more slowly than normal, respectively, in the FGF10-bead based culture model. These data are consistent with the in vivo phenotypes resulted from too little or too much *Spry2* function. A tantalizing speculation, therefore, is that the FGF10-based system could be an invaluable model to readily interrogate aspects of epithelial invasion, including collective migration. Future studies will determine whether these different in vitro systems, when combined with in vivo genetics, could help decipher the cellular and molecular basis underlying epithelial morphogenesis in the mammary gland.

## Supporting Information

Table S1 Primers used in qPCR.
(DOCX)
