# Supplementary material for: Modulation of Fibroblast Growth Factor Signaling Is Essential for Mammary Epithelial Morphogenesis
Source: PLoS One. 2014 Apr 9;9(4):e92735. doi: 10.1371/journal.pone.0092735 (PMC3981693; doi:10.1371/journal.pone.0092735)
Supplement: Table S1 — Primers used in qPCR. (DOCX) [file pone.0092735.s001.docx]

SUPPLEMENTARY TABLE1. Primers used in qPCR.

| Gene name | Forward sequence (5’ 🡪 3’) | Reverse sequence (5’ 🡪 3’) |
| --- | --- | --- |
| *Spry2* | gagaggggttggtgcaaag | ctccatcaggtcttggcagt |
| *Etv4* | ccaccaggatcaagaaggaa | ttgtctgggggagtcatagg |
| *Etv5* | aggaccccaggctgtacttt | tggccgattcttctggatac |
| *Mkp3* | tcgggctgctgctcaagaaac | cggtcaaggtcagactcaatgtcc |
| *Actb* | ggctgtattcccctccatcg | ccagttggtaacaatgccatgt |
| *18S* | gtaacccgttgaaccccatt | gtaacccgttgaaccccatt |
| *EEF1g* | ggattctgtgtgtttgagagca | cagcaaagctgacccactg |
| *GAPDH* | ttcaccaccatggagaaggc | cccttttggctccaccct |
